# Supplementary material for: Causal Relationship between Aging and Anorexia Nervosa: A White-Matter-Microstructure-Mediated Mendelian Randomization Analysis
Source: Biomedicines. 2024 Aug 16;12(8):1874. doi: 10.3390/biomedicines12081874 (PMC11351342; doi:10.3390/biomedicines12081874)
Supplement: Supplementary file 1 [file biomedicines-12-01874-s001.zip › Supplementary Figure/Figure S4/Singe SNP plot of MR analysis for TL to ubm-b-1786.pdf]

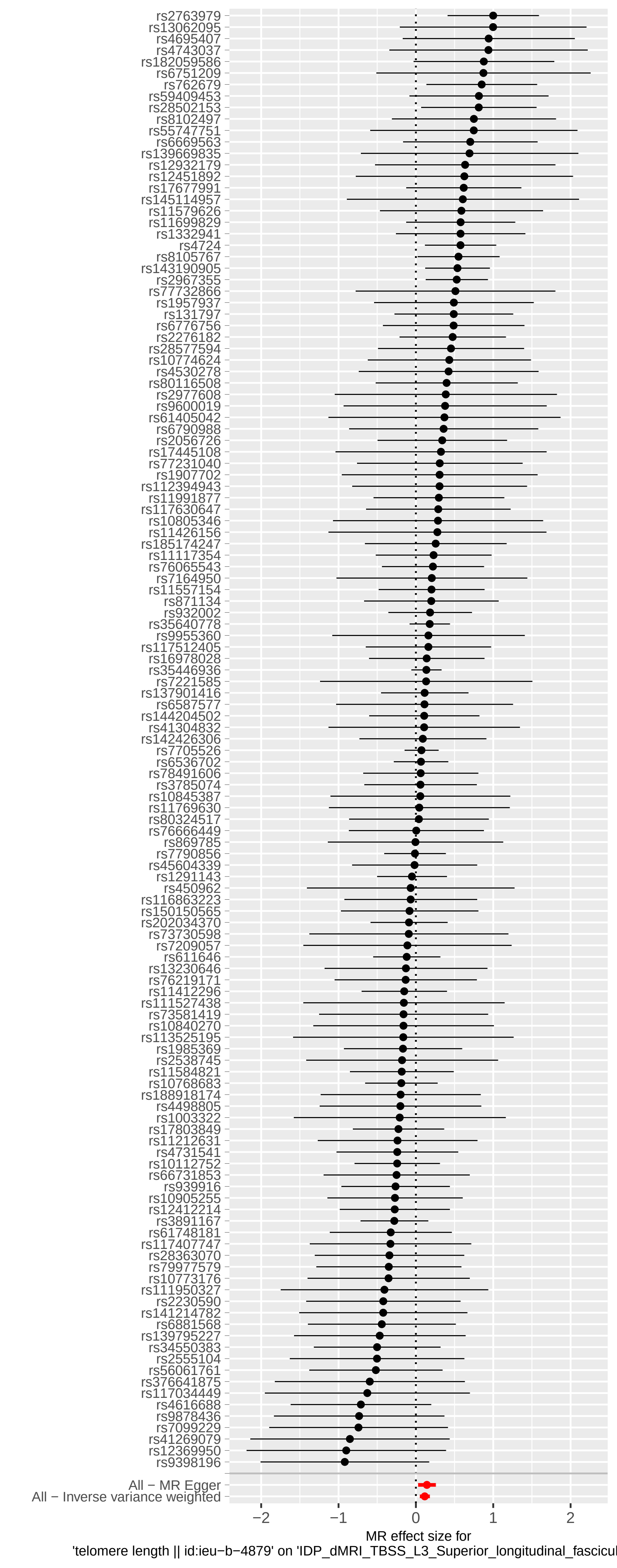
rs2763979
rs13062095
rs4695407
rs4743037
rs182059586
rs6751209
rs762679
rs59409453
rs28502153
rs8102497
rs55747751
rs6669563
rs139669835
rs12932179
rs12451892
rs17677991
rs145114957
rs11579626
rs11699829
rs1332941
rs4724
rs8105767
rs143190905
rs2967355
rs77732866
rs1957937
rs131797
rs6776756
rs2276182
rs28577594
rs10774624
rs4530278
rs80116508
rs2977608
rs9600019
rs61405042
rs6790988
rs2056726
rs17445108
rs77231040
rs1907702
rs112394943
rs11991877
rs117630647
rs10805346
rs11426156
rs185174247
rs11117354
rs76065543
rs7164950
rs11557154
rs871134
rs932002
rs35640778
rs9955360
rs117512405
rs16978028
rs35446936
rs7221585
rs137901416
rs6587577
rs144204502
rs41304832
rs142426306
rs7705526
rs6536702
rs78491606
rs3785074
rs10845387
rs11769630
rs80324517
rs76666449
rs869785
rs7790856
rs45604339
rs1291143
rs450962
rs116863223
rs150150565
rs202034370
rs73730598
rs7209057
rs611646
rs13230646
rs76219171
rs11412296
rs111527438
rs73581419
rs10840270
rs113525195
rs1985369
rs2538745
rs11584821
rs10768683
rs188918174
rs4498805
rs1003322
rs17803849
rs11212631
rs4731541
rs10112752
rs66731853
rs939916
rs10905255
rs12412214
rs3891167
rs61748181
rs117407747
rs28363070
rs79977579
rs10773176
rs111950327
rs2230590
rs141214782
rs6881568
rs139795227
rs34550383
rs2555104
rs56061761
rs376641875
rs117034449
rs4616688
rs9878436
rs7099229
rs41269079
rs12369950
rs9398196
All − MR Egger
All − Inverse variance weighted
−2
−1
0
1
2
MR effect size for
'telomere length || id:ieu−b−4879' on 'IDP_dMRI_TBSS_L3_Superior_longitudinal_fascicul
